# Amelioration of aflatoxin acute hepatitis rat model by bone marrow mesenchymal stem cells and their hepatogenic differentiation

**DOI:** 10.14202/vetworld.2022.1347-1364

**Published:** 2022-05-27

**Authors:** Faten A. M. Abo-Aziza, Abdel Kader A. Zaki, Rana M. Adel, Ahmed Fotouh

**Affiliations:** 1 Department of Parasitology and Animal Diseases, Veterinary Research Institute, National Research Centre, Cairo, Egypt; 2 Department of Physiology, Faculty of Veterinary Medicine, Cairo University, Giza, Egypt; 3 Department of Veterinary Medicine, College of Agriculture and Veterinary Medicine, Qassim University, Buraydah, Saudi Arabia; 4 Zoology Department, Faculty of Women for Arts, Science and Education, Ain Shams University, Cairo, Egypt; 5 Department of Pathology and Clinical Pathology, Faculty of Veterinary Medicine, New Valley University, El-Kharga, Egypt

**Keywords:** acute hepatitis, bone marrow mesenchymal stem cell, cytokines, hepatogenic differentiation, rat model

## Abstract

**Background and Aim:**

Bone marrow-derived mesenchymal stem cells (BM-MSCs) transplantation and their hepatogenic differentiated cells (HDCs) can be applied for liver injury repair by tissue grafting. Regenerative potentiality in liver cirrhosis models was widely investigated; however, immunomodulation and anti-inflammation in acute hepatitis remain unexplored. This study aimed to explore the immunomodulatory and evaluate twice intravenous (IV) or intrahepatic (IH) administration of either BM-MSCs or middle-stage HDCs on aflatoxin (AF) acute hepatitis rat model.

**Materials and Methods:**

BM-MSCs viability, phenotypes, and proliferation were evaluated. Hepatogenic differentiation, albumin, and mmmmmmmm-fetoprotein gene expression were assessed. AF acute hepatitis was induced in rats using AFB1 supplementation. The transplantation of BM-MSCs or their HDCs was done either by IV or IH route. Hepatic ultrasound was performed after 3-weeks of therapy. Cytokines profile (tumor necrosis factor-α [TNF-α], interleukin [IL]-4, and IL-10) was assessed. Hepatic bio-indices, serum, and hepatic antioxidant activity were evaluated, besides examining liver histological sections.

**Results:**

Acute AFB1 showed a significant increase in TNF-α (p<0.01), liver enzyme activities (p<0.05), as well as decrease in IL-4, IL-10, and antioxidant enzyme activities (p<0.05). Cytokines profile was ameliorated in groups treated with IV and IH BM-MCs, showed a negative correlation between IL-4 and TNF-α (p<0.05), and a positive correlation between IL-10 upregulation and TNF-α (p<0.01). In IV HDCs treated group, positive correlations between IL-4 and IL-10 downregulation and TNF-α were observed. However, in IH HDCs group, a significant positive correlation between IL-4 and IL-10 upregulation and TNF-α, were recorded (p<0.05). In addition, IV BM-MSCs and IH HDCs treatments significantly increased antioxidant enzymes activity (p<0.05). IV and IH BM-MSCs significantly ameliorated liver transaminase levels, whereas IH HDCs significantly ameliorated alanine aminotransferase activity and nitric oxide concentration (p<0.05).

**Conclusion:**

The administration routes of BM-MSCs did not demonstrate any significant difference; however, the IH route of HDCs showed significant amelioration from the IV route. On the other hand, it showed noticeable anti-inflammatory and immunomodulatory improvements in aflatoxicosis rats. Therefore, it can be concluded that acute hepatitis can be treated by a noninvasive IV route without the expense of hepatogenic differentiation. Further research using clinical trials that address several problems regarding engraftment and potentiation are needed to determine the optimal manipulation strategy as well as to achieve better long term effects.

## Introduction

Many hepatotoxic agents, including viruses, medications, and toxins, can lead to acute liver injury due to the apoptosis of huge hepatic cells [1]. Aflatoxins (AFs) are secondary metabolites produced by fungal *Aspergillus* species, commonly *Aspergillus flavus* [2]. These fungi grow well in farm-to-table food with the aid of tropical climates [2,3]. As approximately 18 types of AFs are known [4], AFB1 is considered the most widespread toxin [5]. AFB1 affects many tissues, but the liver is the characteristic organ, in which it has been reported to cause hepatocellular malformation after long-term exposure [6]. It is known for its “short-term intoxication” that targets the liver and is marked by acute hepatic failure, degeneration, apoptosis, necrosis, bile duct proliferation, and even death on exposure for a brief period and at high dosage. AFB1 has been reported to interfere with the immune system of both humans and animals. Several studies have recorded a decline in the immunomodulatory factors following AFB1 exposure, such as the decrease in the activity of T or B lymphocytes [7], suppression of macrophage or neutrophil effector functions [8], and modification of inflammatory cytokine synthesis [9], thereby impairing natural killer cells; and increasing risk to diseases. Therefore, understanding the events accompanying short-term exposure to AFB1 is necessary. In spite of the availability of many physical and chemical approaches, they are still restricted along with inadequate effectiveness and cost inferences [10]. These limitations highlight the importance of searching for other alternative approaches [11]. Liver transplantation is a highly effective strategy in several conditions, but lack of donors, high costs required, and rejection of the transplanted organ limit its application [12]. Hepatocyte transplantation could be an alternative to improve liver regeneration; however, there is a deficiency of *in vitro* high-quality principal hepatocytes due to the difficulty of their *in vitro* expansion and the ease of losing their hepatic properties [13]. Hence, liver transplantation and hepatocyte transplantation are inhibited by the scarcity of tissue and cell resources. As a solution, mesenchymal stem cells (MSCs) and their derivatives, including hepatogenic differentiated cells (HDCs) can be used for non-AF liver injury repair in individuals or animal models by grafting into liver tissue, differentiating as well as secreting anti-inflammatory and antioxidant factors, and enhancing *in vivo* liver regeneration as reported by Hu and Li [1]. Several lines of evidence have shown that MSCs and HDCs are useful tools in regenerative medicine that targets liver injury [14].

MSCs can be purified from numerous tissues, including bone marrow (BM), umbilical cord blood and tissue, adipose tissue, and amniotic fluid [15]. MSCs are known for their self-renewal and multipotency and can differentiate into numerous somatic cells, such as osteocytes, chondrocytes, adipocytes, and hepatocytes, according to Hu and Li [16]. Yang *et al*. [17] illustrated that *in vivo* transplantation of MSCs is not only restricted to protection from inflammatory damage but also plays a pivotal function in tissue repair. Research has shown that injured or healthy hepatic conditions affect the response of MSCs, as indicated by the significant reduction in mortality from acute liver injury induced by carbon tetrachloride in mice after allogeneic MSCs implantation into affected tissues, while fewer MSCs were grafted into the normal liver [18]. Thus, MSCs have been proven to be effective for the treatment of liver failure in both animal models and clinical trials, as documented by Kholodenko *et al*. [19]. However, research has shown that engrafted MSCs rarely undergo hepatogenic differentiation at the sites of liver injury and are efficiently cleared from the liver after 1 month of injection [20]. Moreover, Baertschiger *et al*. [21] found that intrahepatic (IH) injection promises stable engraftment of MSCs undergoing myofibroblast differentiation. Other studies reported that this problem could be overcome by the *in vitro* hepatogenic differentiation of MSCs before *in vivo* transplantation.

On the other hand, BM-derived MSCs (BM-MSCs) can undergo functional HDCs, which are predicted to be a potential source of cells in regenerative medicine, tissue engineering, and effective clinical treatment of chronic hepatic injury [22]. After hepatogenic differentiation, MSCs restore liver function and exert immunomodulatory, anti-inflammatory, antifibrotic, antioxidative, and antiapoptotic effects in hepatocytes [23]. Most previous trials used MSCs or differentiated cells for chronic hepatitis models because of the potentiality of these cells to replace the fibrosed tissues [19]. However, the anti-inflammatory and immunomodulatory properties of MSCs have not been effectively utilized in such models [1], which can be attributed to the fact that *in vivo* acute inflammation effectively enhances cell recruitment, whereas chronic inflammation significantly prevents local cell recruitment. Hence, these anti-inflammatory and immunomodulatory properties could be superior and cleared in acute inflammation. It was clear that regenerative potentiality in liver cirrhosis models was widely investigated; however, immunomodulation and anti-inflammation in acute hepatitis remain unexplored.

This study aimed to clarify the degree of effectiveness of these cells in the treatment using an acute aflatoxicosis rat model. In addition, the effects of two routes of administration of either BM-MSCs or middle-stage HDCs in rats with induced aflatoxicosis were explored to determine the effective administration route and types of MSCs for cell-based therapy.

## Materials and Methods

### Ethical approval

This study was approved by Institutional Animal Care and Use Committee of National Research Centre (Protocol Number: 19/151), Cairo, Egypt.

### Study period and location

This study was conducted from February to September 2021 in National Research Centre, Cairo, Egypt.

### Chemicals

All chemicals were purchased from Sigma-Aldrich (USA).

### Isolation of rat BM-MSCs

The albino rats were procured from animal house of National Research Centre, Egypt. BM-derived all-nucleated cells were isolated from the femurs of healthy albino rats with an average weight of 120 g according to the method of Lee *et al*. [24]. The isolated cells were expanded by culturing at 10×10^6^ cell density into 100-mm culture dishes (Greiner Bio-One, Austria) in an expansion medium consisting of alpha minimum essential medium (Sigma-Aldrich, USA) containing 20% fetal bovine serum, L-glutamine (2 mM), 2-mercaptoethanol (55 μM), and penicillin/streptomycin. The cells were incubated in 5% CO_2_ at 37°C for 2-days; the detached cells were discarded by changing the medium and the adherent cells remained in culture for 2-3 weeks. Several passages were performed by subculturing the colony-forming adherent cells in the expansion medium. Subsequently, BM-MSCs were collected at the third passage (P3) on reaching 80% confluence to be exposed for their characterization and proliferation assays.

### Viability and phenotypic analysis of BM-MSCs

Rat BM-MSCs were detached, collected, and centrifuged at 300 × g for 5 min and then suspended in the expansion medium. The viability of BM-MSCS was evaluated in an aliquot using Trypan blue stain by counting the stained live cells and excluding the unstained dead cells as reported by Al-Mutairi *et al*. [25]. For BM-MSC, surface antigen phenotypic analysis and cell markers were assessed [26]. After being washed twice with phosphate-buffered saline (PBS, PH 7.4; 137-mM NaCl, 2.7-mM KCl, 10-mM Na_2_HPO_4_, and 1.8-mM KH_2_PO_4_) (Lonza, Germany) containing 1% bovine serum albumin (ALB) (Sigma-Aldrich), 0.2×10^6^ cells were stained with anti-CD34, anti-CD45, anti-CD29, anti-CD73, and anti-CD90 antibodies (BD Biosciences, USA). A FACSCalibur flow cytometer (BD Biosciences) was used to examine conjugated cells as well as determine the proportions of positive and negative populations. A negative sample with untreated isotype cells was used as a control.

### Proliferation assay using bromodeoxyuridine (BrdU) integration

The proliferative 80% confluent BM-MSCs (P3) were estimated using the BrdU integration assay kit (Invitrogen, USA). Seeding of cells was performed for 2-3 days at a density of 1×10^4^/well on two-well chambered slides (Nunc Lab Tek, Denmark) followed by a 24 h incubation with diluted BrdU staining solution. The number of positively stained cells and that of total cells were counted in ten consecutive images. The proliferation capacity of BM-MSCs was represented as the proportion of BrdU positive cells to the total nucleated cells as explained by Akiyama *et al*. [27].

### Hepatogenic differentiation of BM-MSCs

HDCs were induced through three stages, namely, conditioning, differentiation, and maturation, as described previously by Khajeniazi *et al*. [28]. BM-MSCs (P3) were seeded on collagen Type I coated flasks (Falcon, USA) with an expansion medium at a density of 5×10^3^ until confluence. Cell conditioning (first stage) was induced by preculturing for 2-days with serum-free Iscove’s Modified Dulbecco’s Medium (IMDM; Gibco, USA) containing 10-ng/mL of basic fibroblast growth factor (bFGF2; ITSI-Biosciences, USA) and 20-ng/mL of epidermal growth factor; ITSI-Biosciences. The cells were subjected to 7-days of incubation in IMDM supplemented with 10-ng/mL bFGF2, 20-ng/mL hepatocyte growth factor (HGF; ITSI-Biosciences), and 0.61 g/L nicotinamide (Lonza) to induce cell differentiation (second stage). The maturation stage (third stage) was induced by incubation of cells for up to 21-days in IMDM, including 20-ng/mL HGF, 20-ng/mL of oncostatin M (ITSI-Biosciences), 50-mg/mL of insulin transferrin selenium (Lonza), and 1-μM/L dexamethasone (Sigma-Aldrich). The medium was changed twice a week during the second and third stages. HDCs were morphologically and functionally evaluated at 3 time points: On day 7 (end of differentiation stage –to prematuration stage), day 14 (middle of maturation stage), and day 21 (end of maturation stage).

### Characterization of HDCs

#### Morphology under inverted microscope

The morphological changes of the HDCs were monitored at the three different time points of hepatogenic differentiation and maturation under inverted microscope (Olympus, Japan).

### Reverse transcription-polymerase chain reaction (RT-PCR)

The hepatic markers were assessed using RT-PCR for ALB and 〈-fetoprotein (AFP) genes on day 21 (end of maturation stage) by amplifying the complementary DNA with specific primers as described in Table-1.

**Table 1:** Primers used in the study.

Gene	Primer	Accession number	Amplicon size (bp)
ALB	GGCACAGTGCTTGCAGAATTTCAG (F) CACAGACGGTTCAGGATGGCAG (R)	NM-134326.2	272 bp
AFP	TCTGAAACGCCATCGAAATGCC (F) AATGTAAATGTCGGCCAGTCCCT (R)	NM-012493.2	285 bp

The cells were harvested and stored at −20°C on RNA LATER™ (Thermo Scientific, Germany) to protect cell RNA for further extraction for detection of ALB and AFP gene expression. According to standard protocols; the total cellular RNA was extracted using RNeasy Protect Cell Mini Kit (Invitrogen, catalog no. 12183018A). cDNA synthesis was performed for equal quantities of total RNA in all samples using the Revert-aid first strand cDNA Synthesis Kit (Invitrogen, catalog no.12594025) according to the manufacturer’s protocol. The optimal conditions for denaturation, annealing, and extension steps were carried out using a thermal cycler (Bio-Rad, USA). Five minutes of denaturation were performed at 94°C with 28 cycles for AFP; the denaturation was performed at 94°C for 15-s with 40 cycles for ALB. The annealing and extension were performed at 66°C for 10-s and at 72°C for 20-s, respectively, followed by 7 min of final extension at 72°C. Electrophoresis on agarose gels was used for the separation of PCR products. Relative quantification was normalized against the housekeeping gene glyceraldehyde-3-phosphate dehydrogenase as an internal control and according to Xu *et al*. [29].

### Metabolic activity of HDCs

The concentrations of ALB and urea in the culture media were used as indices for the metabolic function of HDCs on day 7 (end of differentiation stage), day 14 (middle of maturation stage), and day 21 (end of maturation stage) of hepatogenic induction. Urea assay was performed by incubating HDCs in a medium containing 5-mmol/L of ammonium chloride in 5% CO_2_ at 37°C. After 24-h, the supernatant was collected to measure urea concentration colorimetrically using a urea assay kit (BioMerieux, France) according to the manufacturer’s directions. ALB concentrations were determined in the collected media using a bromocresol green colorimetric assay (BioMerieux) following the manufacturer’s directions.

### Injection of BM-MSCs and middle-stage HDCs

BM-MSCs (P3) were used to reach 80% confluence. Middle-stage HDCs were used. Two routes of administration were used in rats after anesthesia was induced with xylazine at 0 mg/kg. An intravenous (IV) injection was performed after washing the tail with xylol for further protrusion of the four tail veins. A suspension of 2×10^6^ cells in 0.5 mL of PBS was prepared and injected slowly into two tail veins. IH injection was performed by determining the injection site on the upper right quadrant of the liver along the left rib edge. Rats were anesthetized with xylazine at 10 mg/kg, and their skin was sterilized with 70% alcohol and shaved before injection. A suspension of 2×10^6^ cells in 0.5 mL PBS was prepared and injected into the liver over 15 min with a syringe at a 30° angle. After injection in both IV and IH routes, gentle pressure was applied on the site of injection using cotton balls to prevent draining and subsequent leakage of the cell suspensions.

### Experimental design

The study was carried out on 48 adult male albino rats weighing 120-150 g. The rats were fed with a standard pellet diet, given water *ad libitum* and kept in standard environmental conditions. They were equally divided into six groups: The first group was supplemented with olive oil and used as control, whereas the other five groups were supplemented with 100 µg/kg body weight AFB; (Sigma-Aldrich) dissolved in olive oil once daily for 7 days [30]. The experimental rats were divided as follows: In Group 2, each rat was injected with 0.5 mL PBS (AFB1 control) (half of the animals received IV injections, whereas the other half received IH injections). In Group 3, each rat was injected with 2×10^6^ BM-MSCs into the tail veins twice at 2-week intervals (IV BM-MSCs). In Group 4, each rat was injected intrahepatically with 2×10^6^ BM-MSCs at 2-week intervals (IH BM-MSCs). In Group 5, each rat was injected with 2×10^6^ middle-stage HDCs into the tail veins twice at 2-week intervals (IV middle-stage HDCs). In Group 6, each rat was injected intrahepatically with 2×10^6^ middle-stage HDCs twice at 2-week intervals (IH middle-stage HDCs). Three weeks after the last injection, all rats were subjected to ultrasonography (USG) examination. Blood samples were collected from all rats and sera were extracted through 3000-rpm centrifugation for 20 min. All rats were humanely killed by cervical dislocation. The guidelines of the National Centre for the Replacement Refinement and Reduction of Animals in Research were followed by the authors, who are well trained in animal care and handling. Animal welfare was taken into consideration to minimize the rats’ discomfort and distress, with sodium pentobarbital anesthesia used during the experiment. Body weight, temperature, and behavioral changes were used as specific endpoint criteria and were monitored daily throughout the experiment. Immediate euthanization was performed when any animal reached the endpoint criteria. Specimens of the liver were dissected from each rat for histological and tissue antioxidant activities analyses.

### USG examination

The rat livers were detected using USG after 3 weeks of therapy [31]. Scanning of the liver was performed using a Doppler USG scanner equipped with a 12 MHz linear array transducer. Gray color and spectral modes were used. All scanning parameters, such as gain, the field of view, and time gain control, were independently optimized. Details of the location, shape, boundaries, and architecture of liver lesions were noted. Color and power flow modes were used to quantify the hepatic blood flow end foci. The peak systolic velocity, end-diastolic velocity (EDV), time average maximum velocity (TAMV), mean velocity (V), systolic/diastolic flow (S/D), resistance index (RI), pulsatility index (PI), G mean, and G peak were measured.

### Cytokine profile

Assay of cytokines levels in the collected rats’ sera was performed using ELISA kits for tumor necrosis factor-α (TNF-α) (catalog no ERT2010-1, Assaypro, USA), interleukin (IL)-4 (RAB0301, Sigma, USA and IL-10 (catalog no ERI3010-1, Assaypro, USA). The manufacturer’s directions were performed, and the color difference was determined at 450 nm using a microplate ELISA reader (BIO-TEK, INC., ELx, 800UV, USA).

### Hepatic bio-indices

Levels of serum total protein, ALB, cholesterol (Chol), alanine aminotransferase (ALT), aspartate aminotransferase (AST), alkaline phosphatase (ALP), triglyceride (TG), nitric oxide (NO), and malondialdehyde (MDA) were measured calorimetrically using kits of (BioMerieux). Globulin and albumin/globulin (A/G) ratio were calculated. The dissected liver samples were weighed and homogenized in PBS (pH 7.4) to yield 20% (w/v) homogenate [32]. Cooling centrifugation was performed at 4°C for 10 min at 1700 rpm. MDA was determined in the supernatant to exhibit lipid peroxidation in the liver.

### Hepatic and serum antioxidant activities

The supernatant of liver homogenate was further diluted with PBS to 2% dilution. Hepatic glutathione peroxidase (GSH-Px), catalase (CAT), superoxide dismutase (SOD), and reduced GSH activities were then determined using SPECTRUM kits (BioMerieux). The absorbencies were estimated spectrophotometrically at 450 nm. Total antioxidant capacity (TAC) and GSH activities were evaluated in the collected sera using SPECTRUM kits (BioMerieux). Absorbencies were spectrophotometrically measured at 340 nm, 560 nm, and 520 nm, respectively.

### Histological assay

Specimens of the collected livers were subjected to histological analysis. Five micro milliliters thickness paraffin slides were processed, followed by hematoxylin-eosin stain to evaluate the amelioration of inflammatory lesions as explained by Bancroft and Marilyn [33].

### Statistical analysis

Statistical Package for the Social Sciences 19.0 was used for statistical analysis. Descriptive statistics and simple one-way analysis of variance were performed. The descriptive values of data were expressed as mean±standard error. *Post hoc* analysis using the Mann–Whitney test was performed to compare two of the six groups and, with p<0.05 and p<0.01 reflecting a significant difference. Minitab 21.0 was used to determine the coefficients of correlations between TNF-α and the bio-index parameters, antioxidant activities, and cytokine profiles.

## Results

### Isolation and proliferation of BM-MSCs

**Figure-1:**
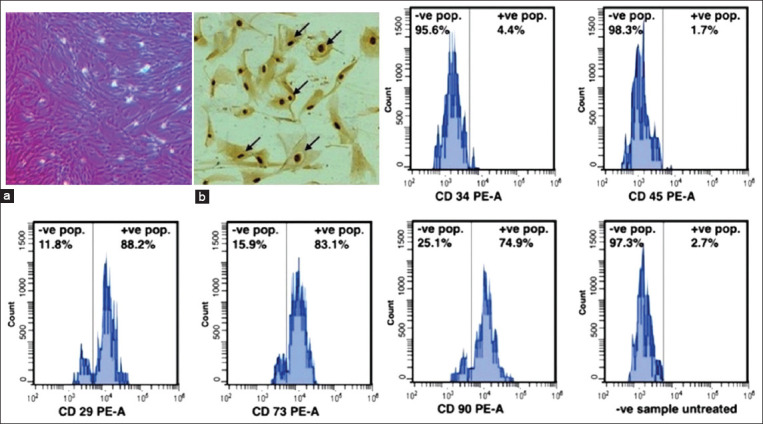
Morphology, proliferation and characterization of bone marrow-derived mesenchymal stem cells (BM-MSCs) at third passage. (a) Photomicrographically image illustrated the 1ry layer of adhered rat BM-MSCs at 80% confluence. (b) Bromodeoxyuridine (BrdU) incorporation assay of 80% confluent BM-MSCs for 24 h showed BrdU +ve cells with a brown stained nucleus (black arrows), ×20. Flow cytometric analyses of BM-MSCs CD markers showed +ve stained cells percentages. BM-MSCs negatively expressed CD34 (4.4%) and CD45 (1.7%) markers while positively expressed CD29 (88.2%), CD73 (83.1%), and CD90 (74.9%) markers.

BM-MSCs were regularly observed by an inverted microscope (Optika, Italy), and they continued to multiply to reach 80% confluence after 14 days (Figure-1a). Trypan blue examination showed that cell viability at the third passage was 97.11±4.08% on reaching 80% confluence. The percentage of BrdU +ve cells with brown stained nucleus was 72.14 (Figure-1b). Flow cytometric analysis showed the percentages of +ve stained cells that indicated the marker of mesenchymalization of isolated cells (Figure-1). Results revealed that BM-MSCs negatively expressed CD34 (4.4%) and CD45 (1.7%) markers while positively expressed CD29 (88.2%), CD73 (83.1%), and CD90 (74.9%) markers (Figure-1).

### Morphological changes of HDCs

**Figure-2:**
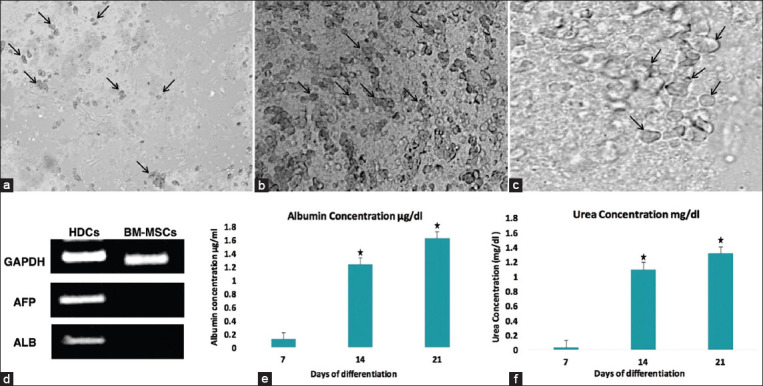
Morphological changes of rat HDCs at different stages of differentiation. (a) The cells were changed from their original cylindrical shape to spherical-shaped cells (arrows) on day 7 (×20). This morphology changed to oblateness (arrows) often in cells from (b) day 14 (×20) to be more compact and polygonal shapes like (arrows) (c) on day 21 of maturation (×40). (d) Reverse transcriptase PCR analysis of hepatic cell genes expression by HDCs and BM-MSCs. Hepatic-associated genes ALB and AFP mRNA were detected on day 21 by HDCs and undetected by BM-MSCs. The housekeeping gene glyceraldehyde-3-phosphate dehydrogenase was used as an internal control. (e) ALB secretion and (f) urea production of HDCs at different time points were estimated on days 7, 14, and 21. Bars with mark (*) were significant than the values on day 7. Error bars represent mean±SE. BM-MSCs=Bone marrow mesenchymal stem cells, HDCs=Hepatogenic differentiated cells, ALB=Albumin, AFP=α-fetoprotein.

The morphology of BM-MSCs was changed from spindle-shaped to oblateness after differentiation to HDCs (Figure-2). The cells were developed from their original cylindrical shape to spherical-shaped cells on day 7 (Figure-2a). This morphology changed to oblateness often in cells from day 14 (Figure-2b) to be more compact, polygonal shapes like on day 21 of maturation of HDCs (Figure-2c). Hepatic specific markers measurement indicated that the protein expression of ALB and AFP (Figure-2d) could be detected as measured by RT-PCR on day 21 of maturation of HDCs. +ve linear correlation of ALB secretion and urea synthesis was obtained by the progress of differentiation. The rates of ALB were significantly higher (p<0.01) on days 14 (1.237±0.14 µg/dL) and day 21 (1.601±0.18 µg/dL) than on day 7 (0.102±0.03 µg/dL) (Figure-2e). Similarly, urea synthesis (Figure-2f) was significantly higher (p<0.01) at day 14 (1.024±0.19 mg/dL) and day 21 (1.136±0.30 mg/dL) than that at day 7 (0.013±0.002) mg/dL) (Figure 2).

### USG examination

#### Hepatic parenchyma

**Figure-3:**
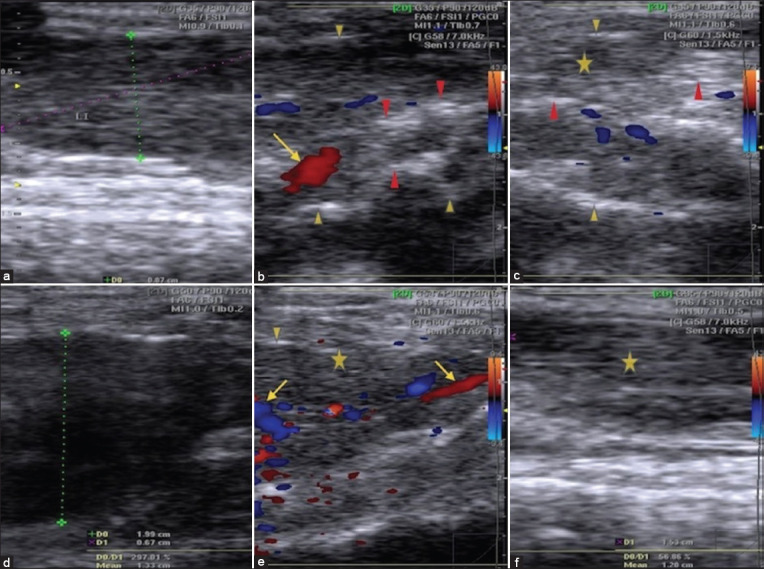
USG of hepatic parenchyma in healthy, acute aflatoxicosis rat model and other different treated groups. (a) Healthy control rat liver. (b) Liver of acute aflatoxicosis rat model. (c) Liver of IV BM-MSCs injected rats at the 4^th^ week. (d) At the 4^th^ week IH BM-MSCs injection. (e) Liver USG at the 4^th^ week following IV HDCs injection. (f) At the 4^th^ week following IV HDCs injection. IV=Intravenous, IH=Intrahepatic, BM-MSCs=Bone marrow mesenchymal stem cells, HDCs=Hepatogenic differentiated cells, USG=Ultrasonography. Marks: Cursor=Surface of liver, arrows=Dilated blood vessels, red arrowheads=Starry sky appearance, star=Parenchyma, yellow arrowheads=Irregular surface of the liver line.

The hepatic parenchyma of healthy control rat livers showed medium echogenicity, with a regular homogeneous surface according to USG (Figure-3a). By contrast, acute aflatoxicosis rat model livers showed enlargement and congestion with a starry sky appearance throughout the hyperechogenic heterogeneous parenchyma, dilated hepatic veins, and an irregular surface line (Figure-3b). Moreover, in the 4^th^ week after injection, USG revealed that the liver of IV BM-MSCs-injected rats had a starry sky appearance throughout the hyperechogenic homogeneous parenchyma (Figure-3c), whereas that of IH BM-MSCs injected rats had a normal lobe with a hypoechogenic homogeneous parenchyma and a regular surface line (Figure-3d). Moreover, USG at 4 weeks after IV HDCs injection showed a congested liver with a hyperechogenic parenchyma and a slightly irregular liver surface line (Figure-3e). Conversely, USG at 4 weeks after IH HDCs injection demonstrated an improvement as shown by a medium echogenic homogeneous hepatic parenchyma and a regular surface line (Figure-3f).

### Hepatic blood flow

**Figure-4:**
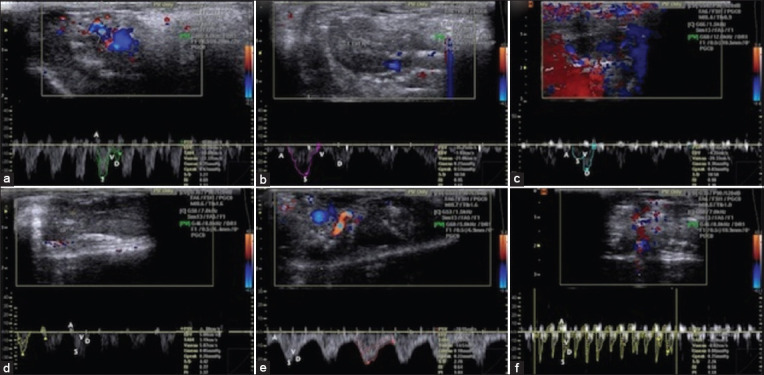
Color and spectral Doppler USG of hepatic venous flow direction and waveform in healthy, acute aflatoxicosis rat model, and other different treated groups. (a) Hepatic venous flow direction and waveform images in healthy control rats; the hepatic venous waveform was phasic, and the direction of flow was predominantly antegrade. (b) By contrast, in the acute aflatoxicosis rat model, the hepatic venous waveform was phasic, and the direction of flow was antegrade corresponding to the three waves (A, S, and D). Abnormally mild decreased phasicity was observed in the hepatic vein of the acute aflatoxicosis rat model. (c) In the 4^th^ week after IV BM-MSCs injection of rats, the hepatic venous waveform showed phasicity and the direction of blood flow was antegrade that corresponding to the three waves (A, S, and D) through color and spectral Doppler USG. (d) Conversely, in the 4^th^ week after IH MSCs injection of rats, normal hepatic venous flow direction and waveform were observed: The hepatic venous waveform was phasic and predominantly antegrade with a tetrainflectional waveform (A, S, V, and D waves) that was mostly below the baseline in spectral Doppler USG. (e) The IV HDCs injected rats showed a phasic waveform with an antegrade direction of hepatic venous flow, which corresponded to the three waves (A, S, and D) through color and spectral Doppler USG, at 4 weeks after injection. (f) By contrast, normal hepatic venous flow direction and waveform were observed via color and spectral Doppler USG at 4 weeks after injection in the IH HDCs injected rats: The hepatic venous waveform was phasic and predominantly antegrade with tetrainflectional waveform. IV=Intravenous, IH=Intrahepatic, BM-MSCs=Bone marrow mesenchymal stem cells, HDCs=Hepatogenic differentiated cells, USG=Ultrasonography.

Color and spectral Doppler USG results showed normal hepatic venous flow direction and waveform picture in healthy rats in which the hepatic venous waveform was phasic, and the direction of flow was predominantly antegrade. A tetra-inflectional waveform (A, S, V, and D waves) was observed that was mostly below the baseline at spectral Doppler. The A wave was an upward pointing with a peak corresponding to the maximum retrograde hepatic venous flow above the baseline. The S wave was the next wave on the waveform. Its initial downward-sloping resembles antegrade hepatic venous flow and considered the largest downward-pointing wave in the cycle. The v wave was the third wave on the waveform. The D wave was the 4^th^ and the smaller last downward-pointing wave that attributed to antegrade hepatic venous flow. The distance between the A wave and peak −ve excursion S wave was normal referring to normal phasicity (Figure-4a). However, in acute aflatoxicosis rat model, the hepatic venous waveform was phasic, and the direction of flow was antegrade that corresponded to the three waves (A, S, D). Abnormally mild decreased phasicity was noticed in the hepatic vein of acute aflatoxicosis rat model. This low phasicity was attributed to mild decrease in the distance between the A wave and peak −ve S wave excursion as shown in the spectral Doppler USG (Figure-4b). At the 4^th^ week following IV MSCs injection of rats, the hepatic venous waveform showed phasic, and the direction of blood flow was antegrade that corresponded to the three waves (A, S, and D) by Color and Spectral Doppler USG. In addition, a pulsatile waveform of hepatic vein was observed but the *S* wave was not as deep as the D wave leading to with “decreased *S* wave” that was specific for tricuspid regurgitation (Figure-4c). However, Color and spectral Doppler USG illustrated normal hepatic venous flow direction and waveform at the 4^th^ week in IH MSCs injected rats. The hepatic venous waveform was phasic and predominantly antegrade with tetrainflectional waveform (A, S, V, and D waves) that was mostly below the baseline at spectral Doppler. Particularly, the A wave was upward-pointing wave with a peak of maximum retrograde hepatic venous flow. The S wave showed downward-sloping corresponding to antegrade hepatic venous flow. The v wave was the third wave on the waveform. The D wave was the 4^th^ and last wave and corresponded to antegrade hepatic venous flow as well as it was the smaller of the two downward-pointing waves. The distance between the A wave and peak −ve excursion S wave was normal referring to normal phasicity (Figure-4d). On the other hand, IV HDCs injected rats showed phasic waveform with antegrade direction of hepatic venous flow which corresponded to the three waves (A, S, and D) by Color and Spectral Doppler USG at the 4^th^ week of injection. Abnormally moderate decreased phasicity in the hepatic vein was also observed by Spectral Doppler USG as the A wave was downward-pointing wave and the most decreased was the distance between the A wave and the −ve S wave peak (Figure-4e). However, normal hepatic venous flow direction and waveform was observed by Color and spectral Doppler USG at the 4^th^ week in IH HDCs injected rats. The hepatic venous waveform was phasic and predominantly antegrade with tetra-inflectional waveform (A, S, V, and D waves). Particularly, the A wave was upward-pointing with a peak corresponding to maximum retrograde flow of hepatic vein. The S wave was the next wave on the waveform with downward-sloping corresponding to antegrade hepatic venous flow. The v wave was the third wave on the waveform and the D wave was the 4^th^ and last one corresponding to antegrade flow of hepatic vein and is the smaller downward-pointing wave on the waveform. In addition, the distance between the A wave and peak −ve excursion S wave was normal referring to normal phasicity (Figure-4f).

### Blood flow parameters

A picture of high blood flow was noticed in acute AFB1 that represented by elevated PSV, TAMV, PI, G mean and G peak as well as declined EDV, S/D, and RI. Both IV and IH injection of BM-MSCs resulted in a significant decrease of PSV and TAMV and increase of EDV, S/D, and RI in IV BM-MSCs and IH BM-MSCs groups comparing to AFB1 control group. However, no changes appeared after IV injection of middle-stage HDCs. On the other hand, IH injection of middle-stage HDCs led to the decline of PSV, TAMV, and G peak as well as elevation of EDV, S/D, and RI in IH middle-stage HDCs group comparing to AFB1 control group (Table-2).

**Table 2:** Blood flow profile of rats with acute aflatoxicosis and after therapy.

Parameters	Healthy control (n=8)	AFB1 (n=8)	AFB1+IV BM-MSCs (n=8)	AFB1+IH BM-MSCs (n=8)	AFB1+IV HDCs (n=8)	AFB1+IH HDCs (n=8)	p-value
PSV (cm/s)	25.72±1.18	58.36*±6.41	38.41^a^±2.48	32.85^a^±3.76	49.61±3.29	29.95^a^±2.81	0.0032
EDV (cm/s)	9.18±2.03	2.01*±0.42	8.52^a^±1.93	9.78^a^±2.46	3.93±1.05	8.96^a^±1.77	0.0014
TAMV (cm/s)	8.05±0.64	14.25*±1.51	7.08^a^±1.28	8.42^a^±1.13	15.42±3.07	8.93^a^±0.72	0.0011
Mean V (cm/s)	15.62±1.28	19.04±3.38	14.90±2.18	16.48±2.38	18.76±3.58	15.33±1.49	0.0913
S/D	2.74±0.71	0.31*±0.07	1.23^a^±0.25	3.51^a^±1.02	0.66±0.13	0.43^a^±0.05	0.0011
RI	1.83±0.27	0.61*±0.13	1.53^a^±0.22	1.84^a^±0.33	0.76±0.19	1.87^a^±0.39	0.0013
PI	1.02±0.16	2.92*±0.53	1.78±0.37	1.46±0.18	2.79±0.52	1.88±0.33	0.0422
G Mean (mmHg)	0.12±0.03	0.29*±0.04	0.17±0.03	0.24±0.05	0.25±0.05	0.19±0.02	0.0321
G Peak (mmHg)	0.25±0.07	0.73*±0.15	0.39±0.04	0.33±0.08	0.63±0.14	0.26^a^±0.02	0.0824

• In the same row significantly different than control at P<0.01. and

a is significantly different than AFB1 at P*<*0.01. AFB1=Aflatoxin B1, IV=Intravenous, IH=Intrahepatic, BM-MSCs=Bone marrow mesenchymal stem cells, HDCs=Hepatogenic differentiated cells, PSV=Peak systolic velocity, EDV=End diastolic velocity, TAMV=Time average maximum velocity, V=Mean velocity, S/D=Systolic/diastolic flow, RI=Resistance index, PI=Pulsatility index

### Cytokines profile and correlation to TNF-α

AFB1 group was associated with a significant increase of the pro-inflammatory cytokine (TNF-α) and a significant decline in the anti-inflammatory cytokines (IL-4 and IL-10) at p<0.05. IH therapy with HDCs ameliorated the levels of cytokines as indicated by a significant decrease in the serum TNF-α level (p<0.05) and a significant increase of IL-10 level (p<0.05) comparing to AFB1 group (Table-3).

**Table 3:** Cytokines concentration (ng/mL) of rat serum with acute aflatoxicosis and after therapy.

Parameters	Healthy control (n=8)	AFB1 (n=8)	AFB1+IV BM-MSCs (n=8)	AFB1+IH BM-MSCs (n=8)	AFB1+IV HDCs (n=8)	AFB1+IH HDCs (n=8)	p-value
TNF-a	10.99±2.11	15.61*±1.31	11.32±1.91	14.21±2.30	16.87±2.12	10.25^a^±1.63	0.0382
IL-4	12.47±1.33	8.36*±1.54	11.99±2.22	10.52±1.49	7.31±1.47	11.85±1.64	0.0461
IL-10	8.35±1.20	5.85*±1.23	7.32±1.25	7.33±1.86	5.66±1.70	9.07^a^±1.58	0.0419

• In the same row, significantly different than control at P*<*0.01. And

a is significantly different than AFB1 at P*<*0.05. AFB1=Aflatoxin B1, IV=Intravenous, IH=Intrahepatic, BM-MSCs=Bone marrow mesenchymal stem cells, HDCs=Hepatogenic differentiated cells, TNF=Tumor necrosis factor, IL=Interleukin

**Figure-5:**
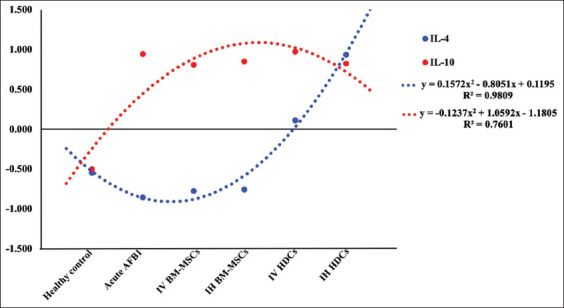
Correlation of serum levels of IL-4 and IL-10 to TNF-α value. AFB1=Aflatoxin B1, IV=Intravenous, IH=Intrahepatic, BM-MSCs=Bone marrow mesenchymal stem cells, HDCs=Hepatogenic differentiated cells, TNF=Tumor necrosis factor, IL=Interleukin.

In AFB1 group, a significant −ve correlation was observed in IL-4 (p<0.05) and a significant downregulation +ve correlation in IL-10 (p<0.01) to TNF-α. The results indicated amelioration of cytokines concentration in IV BM-MSCs and IH BM-MCs treated groups with −ve correlation in IL-4 (p<0.05) and a significant upregulation +ve correlation in IL-10 (p<0.01) to TNF-α. In the IV HDCs treated group, a downregulation +ve correlation was recorded in both IL-4 and IL-10 to TNF-α. However, a significant upregulation +ve correlation was recorded in both IL-4 and IL-10 (p<0.05) to TNF-α of IH HDCs treated group (Figure-5 and Table-4).

**Table 4:** Correlation coefficient values of serum cytokines, liver bio-indices, and antioxidant enzymes to TNF.

Parameters	Healthy control (n=8)	AFB1 (n=8)	AFB1+IV BM- MSCs (n=8)	AFB1+IH BM- MSCs (n=8)	AFB1+IV HDCs (n=8)	AFB1+IH HDCs (n=8)
IL-4	−0.547^a^	−0.855	−0.774	−0.754	0.111	0.938
IL-10	−0.500^b^	0.944	0.808	0.852	0.974	0.826
Chol	0.888	0.900^a^	0.900^a^	1.000^b^	0.951	0.721^a^
ALT	0.849	1.000	0.947	0.900	0.791	1.000
AST	0.999	1.000	0.999^a^	0.999^a^	1.000	−1.000^a^
ALP	0.990	0.996^a^	0.979	0.952	−1.000	0.987
TG	0.999	1.000^a^	0.845	1.000^b^	−0.833	0.934
NO	0.994	0.997	1.000	0.900^a^	−0.751^a^	1.000
MDA	0.956	0.912	1.000^b^	0.999^b^	−0.971^b^	1.000^b^
GSH	0.990	−0.996	−1.000	−0.853^b^	1.000^b^	−0.984^b^
TAC	1.000	−0.766^a^	−1.000^b^	−0.912^b^	0.853	−0.862

Values of correlation coefficient with

a and

b are significantly correlated with TNF-α. AFB1=Aflatoxin B1, IV=Intravenous, IH=Intrahepatic, BM-MSCs=Bone marrow mesenchymal stem cells, HDCs=Hepatogenic differentiated cells, IL=Interleukin, Chol=Cholesterol, ALT=Alanine aminotransferase, AST=Aspartate aminotransferase, ALP=Alkaline phosphatase, TG=Triglyceride, NO=Nitric oxide, MDA=Malondialdehyde, GSH=Reduced glutathione, TAC=Total antioxidant capacity

### Hepatic bio-indices and correlations with TNF-α

AFB1 injection led to alterations in some liver function parameters. A significant decrease in ALB concentration (p<0.05) and a significant increase in ALT, AST (p<0.05), and NO (p<0.01) were observed. IV injection of BM-MSCs significantly ameliorated ALT and AST activities (p<0.05). By contrast, IH injection of BM-MSCs significantly ameliorated ALT (p<0.05), AST (p<0.01), and NO (p<0.01) compared with AFB1 control. IV therapy with HDCs did not affect the bio-index parameters and liver enzyme activities whereas, IH therapy with HDCs significantly increased ALB concentration (p<0.05) and decreased ALT activity (p<0.05) and NO concentration (p<0.01) to approximately normal levels. Serum MDA significantly increased (p<0.01) in the AFB1 group compared with that in the healthy control group. IH injection of HDCs restored the serum level of MDA (p<0.01) (Table-5).

**Table 5:** Hepatic bio-indices parameters and enzyme activities of rat serum with acute aflatoxicosis and after therapy.

Parameters	Healthy control (n=8)	AFB1 (n=8)	AFB1+IV BM - MSCs (n=8)	AFB1+IH BM - MSCs (n=8)	AFB1+IV HDCs (n=8)	AFB1+IH HDCs (n=8)	p-value
Total protein (g/dL)	6.14±1.25	6.44±1.85	6.53±1.44	6.48±1.84	7.01±1.77	6.99±1.08	0.1942
ALB (g/dL)	4.55±1.02	3.18*±0.51	3.15±0.61	3.66±0.88	3.46±0.61	4.11^a^±1.14	0.0421
Globulin (g/dL)	3.45±0.11	3.98±0.24	3.11±0.14	3.60±0.42	3.15±0.32	3.47±0.51	0.0933
A\G ratio	1.2±0.05	0.94±0.04	1.01±0.10	1.01±0.12	1.10±0.11	1.40±0.20	0.5426
Chol (mg/dL)	89.11±11.52	90.34±13.04	85.22±6.75	75.17±8.55	81.05±11.43	89.38±12.42	0.7114
ALT/GPT (U/L)	65.90±4.68	89.07*±6.52	64.11^a^±8.51	61.99^a^±8.01	91.15±9.54	69.03^a^±8.52	0.0030
AST/GOT (U/L)	133.47±12.54	164.12*±14.20	148.25^a^±14.25	131.38^b^±12.55	178.13±14.85	180.24±18.26	0.0061
ALP (U/dl)	124.16±18.58	134.55±19.35	118.62±20.84	124.85±21.77	133.01±18.25	130.84±20.74	0.4430
TG (mg/dL)	31.06±3.55	34.15±4.51	33.84±2.40	31.42±3.42	30.45±5.21	32.14±4.20	0.3216
NO (umol/L)	16.05±3.25	25.48**±2.09	23.84±1.52	14.65^b^±2.87	12.88±3.10	11.65^b^±2.84	0.0241
MDA (nmol/mL)	0.489±0.021	0.981**±0.01	0.882±0.01	0.771±0.01	0.731±0.06	0.431^b^±0.031	0.0077

In the same row,

* and

** are significantly different than control at P*<*0.05 and P*<*0.01 respectively and

a and

b are significantly different than AFB1 at P*<*0.05 and P*<*0.01, respectively. AFB1=Aflatoxin B1, IV=Intravenous, IH=Intrahepatic, BM-MSCs=Bone marrow mesenchymal stem cells, HDCs=Hepatogenic differentiated cells, ALB=Albumin, A/G=Albumin/globulin, Chol=Cholesterol, ALT=Alanine aminotransferase, AST=Aspartate aminotransferase, ALP=Alkaline phosphatase, TG=Triglyceride, NO=Nitric oxide, MDA=Malondialdehyde

**Figure-6:**
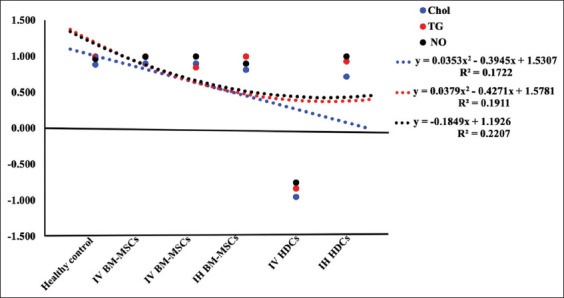
Correlation of serum levels of Chol, TG, and NO to TNF-α value. AFB1=Aflatoxin B1, IV=Intravenous, IH=Intrahepatic, BM-MSCs=Bone marrow mesenchymal stem cells, HDCs=Hepatogenic differentiated cells, Chol=Cholesterol, TG=Triglyceride, NO=Nitric oxide.

**Figure-7:**
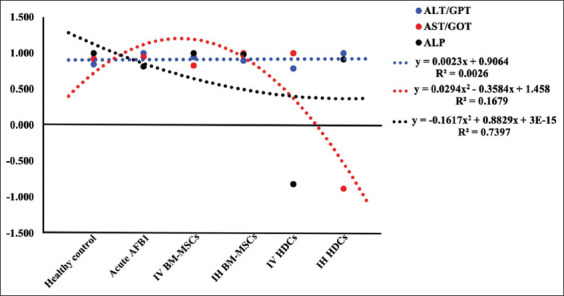
Correlation of serum activities of ALT, AST, and ALP to TNF-α value. AFB1=Aflatoxicosis, IV=Intravenous, IH=Intrahepatic, BM-MSCs=Bone marrow mesenchymal stem cells, HDCs=Hepatogenic differentiated cells, ALT=Alanine aminotransferase, AST=Aspartate aminotransferase, ALP=Alkaline phosphatase, TNF=Tumor necrosis factor.

The level of the bio-indices and enzyme activities in the serum of AFB1 rats correlated with TNF-α (Figures-6 and 7; Table-4). In the AFB1 group, significant +ve correlations were observed between Chol, ALP, and TG upregulation and TNF-α (p<0.05). However, significant −ve correlations were observed between Chol, TG, and NO serum levels and TNF-α (p<0.01) after IV HDCs therapy (Figure-6).

In AFB1, significant +ve correlations of ALT, AST, and ALP upregulation with TNF-α (p<0.05) were recorded. Amelioration of these liver enzymes was observed after treatment with IV and IH BM-MSCs indicating a significant +ve correlation between downregulation and TNF-α (p<0.05). After IV HDCs therapy, significant +ve correlations between ALT and AST downregulation and TNF-α, as well as a significant −ve correlation between ALP serum level and TNF-α were recorded. After IH HDCs therapy, the downregulation of ALT and ALP serum levels was significantly positively correlated with TNF-α (p<0.05), but the AST was significantly negatively correlated with TNF-α (Figure-7).

### Serum antioxidant activities and correlation to TNF-α

In AFB1 group, serum GSH and TAC activities were significantly decreased at (p<0.05) and (p<0.01), respectively, compared to the healthy control group. Treatment with IV injection of BM-MSCs improved the serum activities of GSH and TCA as indicated by a significant increase in their serum levels at (p<0.05) and (p<0.01), respectively. Treatment with IH injection of HDCs restored serum antioxidant activities as accompanied by a significant elevation in serum GSH (p<0.05) comparing to AFB1 group (Table-6).

**Table 6:** GSH and TAC activities in rat serum with acute aflatoxicosis and after therapy.

Parameters	Healthy control (n=8)	AFB1 (n=8)	AFB1+IV BM-MSCs (n=8)	AFB1+IH BM-MSCs (n=8)	AFB1+IV HDCs (n=8)	AFB1+IH HDCs (n=8)	p-value
GSH (nmol/mL)	33.51±2.64	20.18*±3.73	32.86^a^±2.11	27.55±3.09	23.76±1.65	30.33^a^±2.17	0.0125
TAC (mM/L)	1.76+0.24	0.48**+0.03	1.54^b^+0.36	0.76+0.03	0.54+0.08	0.56+0.07	0.0331

In the same row,

* and

* are significantly different than control at P*<*0.05 and P*<*0.01 respectively and

a and

b are significantly different than AFB1 at P*<*0.05 and P*<*0.01, respectively. AFB1=Aflatoxin B1, IV=Intravenous, IH=Intrahepatic, BM-MSCs=Bone marrow mesenchymal stem cells, HDCs=Hepatogenic differentiated cells, GSH=Reduced glutathione, TAC=Total antioxidant capacity

**Figure-8:**
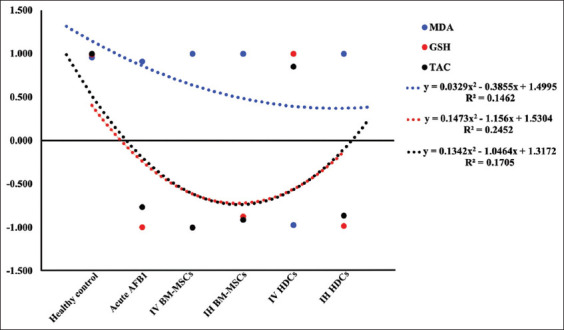
Correlation of serum level and antioxidant activities to TNF-α value. AFB1=Aflatoxin, IV=Intravenous, IH=Intrahepatic, BM-MSCs=Bone marrow mesenchymal stem cells, HDCs=Hepatogenic differentiated cells, MDA=Malondialdehyde, GSH=Reduced glutathione, TAC=Total antioxidant capacity.

In acute the AFB1 rat model, a significant −ve correlation in GSH and TAC serum activities to TNF-α were observed. Furthermore, a significant −ve correlation in GSH and TAC serum activities to TNF-α was observed in IV BM-MSCs, IH BM-MSCs, and IH HDCs treated rats. However, a significant upregulation +ve correlation of GSH and TAC serum activities (p<0.05) to TNF-α was noticed in IV HDCS treated rats (Figure-8).

### Tissue antioxidant enzymes

For the activities of the antioxidant enzymes (GSH-Px, CAT, and SOD) in hepatic tissue, it was noticed that AFB1 injection resulted in a significant decrease in the antioxidant activities of CAT (p<0.01), SOD, GSH, and GSH-Px (p<0.05) compared to the healthy control group. IV therapy with BM-MSCs significantly increased the antioxidant activities of CAT, SOD, and GSH-Px activities (p<0.05). IH therapy of BM-MSCs and IV therapy of HDCs did not ameliorate the toxic effect of AFB1. However, IH therapy of HDCs significantly restored the antioxidant activities of SOD and GSH-Px (p<0.05) without change in CAT activity comparing to AFB1 control group (Table-7).

**Table 7:** Antioxidant enzyme activities and MDA of hepatic tissues of rat with acute aflatoxicosis and after therapy.

Parameters	Healthy control (n=8)	AFB1 (n=8)	AFB1+IV BM-MSCs (n=8)	AFB1+IH BM-MSCs (n=8)	AFB1+IV HDCs (n=8)	AFB1+IH HDCs (n=8)	p-value
CAT (nmol/min/g tissue)	34.60±4.11	21.44**±2.71	31.95^a^±2.15	23.26±2.06	26.19±2.28	28.12±3.08	0.0113
SOD (U/g tissue)	8.11±1.17	5.19*±1.06	7.14^a^±0.35	5.90±1.23	5.06±1.97	8.22^a^±0.97	0.0120
GSH (mM/g tissue)	7.47±1.51	4.95*±0.82	6.66±1.30	4.57±0.87	5.52±0.72	7.01^a^±1.04	0.0141
GSH-Px (U/g tissue)	65.42±5.39	45.22*±3.62	60.22^a^±4.81	48.16±3.25	51.63±5.63	58.31^a^±4.17	0.0139
MDA (nmol/g tissue)	33.85±3.11	35.74±2.15	31.06±2.05	31.83±1.89	32.16±3.77	32.22±2.16	0.4620

In the same row,

* and

** are significantly different than control at P*<*0.05 and P*<*0.01 respectively and

a is significantly different than AFB1 at P*<*0.05. AFB1=Aflatoxin B1, IV=Intravenous, IH=Intrahepatic, BM-MSCs=Bone marrow mesenchymal stem cells, HDCs=Hepatogenic differentiated cells, CAT=Catalase, SOD=Superoxide dismutase, GSH=Reduced glutathione, GSH-Px=Glutathione peroxidase, MDA=Malondialdehyde

### Gross and microscopic examination of liver of rat with acute aflatoxicosis rats and after therapy

**Figure-9:**
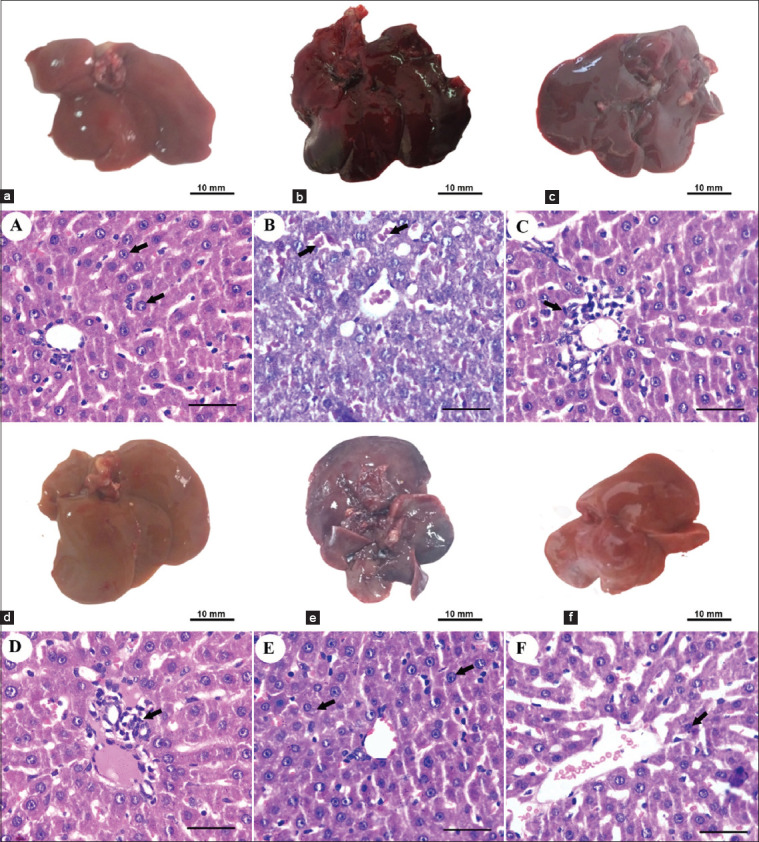
Gross appearance and histological examination of Liver of different rat groups. (a, A) The liver of the healthy control group showed grossly normal appearance and size and the histological examination showed apparently normal hepatocytes. (b, B) In the acute AFB1 control group, the liver showed swollen hepatomegaly with severe congestion and dark borders by gross appearance with extensive necrosis of hepatocytes (arrows) by histopathological examination. (c, C) Liver restored to nearly normal gross appearance and size after 4 weeks in IV BM-MSCs. (d, D) IH BM-MSCs groups with few inflammatory cells infiltration (arrow) in IV BM-MSCs and apparently normal hepatocytes with few mononuclear cells infiltrating the portal area (arrow) in IH BM-MSCs by histological examination. (e, E) In the IV HDCs group, livers appeared swollen, congested, and enlarged and showed apparently normal hepatocytes (arrows) by histopathological examination. (f, F) IH HDCs group showed nearly normal healthy gross appearance of the liver with the disappearance of congestion and showed Kupffer cell hyperplasia (arrow) by histopathological examination. Scale bars=50 μm. AFB1=Aflatoxicosis, IV=Intravenous, IH=Intrahepatic, BM-MSCs=Bone marrow mesenchymal stem cells, HDCs=Hepatogenic differentiated cells.

The gross examination revealed that injection of AFB1 resulted in swollen hepatomegaly with severe congestion and dark borders as well as, the histopathological examination of livers showed extensive necrosis of hepatocytes. Liver restored to nearly normal gross appearance and size after 4 weeks in IV BM-MSCs and IH BM-MSCs groups. In addition, liver histopathological examination showed few inflammatory cells infiltration in IV BM-MSCs and apparently normal hepatocytes with few mononuclear cells infiltrating the portal area in IH BM-MSCs. In the IV HDCs group, livers appeared swollen, congested, and enlarged by gross examination and showed apparently normal hepatocytes by histopathological examination. IH HDCs group showed nearly normal healthy gross appearance of the liver with the disappearance of congestion and showed Kupffer cell hyperplasia (arrowhead) by histopathological examination (Figure-9)

## Discussion

The liver is the principal organ to receive absorbed toxins after oral ingestion; consequently, it is usually subjected to acute hepatitis [34]. Aflatoxicosis is a pathological problem categorized under hepatocellular abnormalities characterized by the infiltration of inflammatory cells, apoptosis, and necrosis. Different scientific approaches have been used to treat liver injuries, but none has been found to be superior. Stem cell transplantation, which is relatively new, is considered to be an effective alternative therapy for hepatic diseases. To date, more than 1000 clinical trials have used MSC therapy in regenerative medicine; of these, approximately 80 clinical trials have targeted liver disease [35]. Most of the trials used liver cirrhosis models, but data on acute hepatitis remain scanty. Although BM-MSCs have the *in vitro* ability to differentiate into functional and morphological liver cells, whether BM-MSC injection or transplantation can alleviate acute degenerated hepatocytes *in vivo* is still controversial. A significant number of recently published papers support that BM-MSCs therapy in different types of hepatitis works through the secretion of soluble suppressive factors through cell-to-cell contact [36] or by a paracrine mechanism that alters the function of the immune cells [37]. In addition to the descriptive problems, the optimal administration route for therapy in animal models with hepatitis remains controversial and requires further investigation to achieve better effects [38].

In this study, BM-MSCs exhibited 97.11%±4.08 viability at the third passage on reaching 80% confluence using Trypan blue stain. The proliferation rate was 72.14% after BrdU incorporation assay. Flow cytometric analysis showed the percentage of positively stained cells that served as the markers of mesenchymalization of isolated cells. The results revealed that BM-MSCs negatively expressed CD34 (4.4%) and CD45 (1.7%) markers while positively expressed CD29 (88.2%), CD73 (83.1%), and CD90 (74.9%). These data substantiate the use of isolated cells to complete the experiments as indicated previously by Abo-Aziza *et al*. [39] and Zaki *et al*. [40].

*In vitro* hepatogenic differentiation of BM-MSCs indicated their transformation from spindle to oblate. The cells developed from their original cylindrical shape into spherically shaped cells on day 7. This morphological change progressed to oblateness on day 14 to be more compact and to a pseudo-polygonal shape on day 21 of maturation of HDCs. Hepatic specific marker measurement indicated that the protein expression of ALB and AFP could be measured through RT-PCR on day 21 of maturation of HDCs. The functional properties of ALB secretion and urea synthesis were analyzed to evaluate the hepatic specific function of HDCs. A +ve linear correlation between ALB secretion and urea synthesis was determined by the progress of differentiation. The rates of ALB secretion and urea synthesis were significantly higher on days 14 and 21 than they were on day 7. These results indicated that ALB secretion and urea synthesis increased significantly with the development of differentiation steps to reach the maximum at the maturation of HDCs. These data are consistent with those of the previous studies on the expression of ALB and urea [41] as well as that of AFP [29]. Hepatocyte like cells (HLCs) have multiple hepatic functions, such as releasing ALB and AFP and promoting the regeneration of multiple liver cells by declining the degeneration process, as indicated by Chetty *et al*. [42].

Acute AFB1 toxicity was associated with a significant increase in TNF-α as well as a decrease in IL-4 and IL-10. IH therapy with HDCs resulted in a significant decrease in the serum TNF-α level and a significant increase in the IL-10 level. In the AFB1 group, a significant −ve correlation between IL-4 and TNF-α and a significant +ve correlation between IL-10 downregulation and TNF-α were observed. These results indicated amelioration of cytokine concentration in the IV and IH BM-MCs treated groups with a −ve correlation between IL-4 and TNF-α as well as a significant +ve correlation between IL-10 upregulation and TNF-α. In the IV HDCs group, +ve correlations between IL-4 and IL-10 downregulation and TNF-α were recorded. By contrast, in the IHDCs group, significant +ve correlations between both IL-4 and IL-10 upregulation and TNF-α were recorded. In a previous study, MSCs reduced liver injury and lowered IL-1β, IL-6, and TNF-α levels in a lipopolysaccharide-induced acute liver failure rat model [15]. A study conducted by Teshima *et al*. [43] reported that transplantation of MSCs isolated from adipose tissue through the peripheral vein decreased the levels of IL-1, IL-6, IL-8, and interferon γ (pro-inflammatory cytokines) and increased the levels of IL-4 and IL-10, HGF, and vascular endothelial growth factor (anti-inflammatory cytokines) in an acute liver failure animal model, whereas transplantation through the splenic vein significantly increased the number of MSCs engrafted in the liver compared with transplantation via the peripheral vein. IV injection has been established as the most suitable administration route for improving liver function and decreasing TNF-α [44]. Different types of cytokines, including TNF-α; in addition to the infiltration of leukocytes, are released by inflamed hepatic tissues and effectively promote the migration of MSCs into the site of injury, as reported by Squillaro *et al*. [45]. However, another study reported that intraportal injection was superior to tail vein injection for improving the therapeutic effects of MSCs on liver dysfunction [46]. The possible mechanisms by which stem cells decrease the proliferation of anti-inflammatory cytokines and T cells are through cell-to-cell contact and paracrine mechanisms, as discussed previously by Hu *et al*. [47]. MSCs have been reported to produce prostaglandin E_2_, resulting in increased production of IL-10 as an anti-inflammatory cytokine and diminished production of TNF-α, interferon γ, and IL-12 [48]. In the same way, transplantation of HLCs eliminated CCl4-induced liver fibrosis and conserved the function of the liver via the release of transforming growth factors β 1, IL-6, and IL-10 [49].

Dose and repetition of MSC transplantation also affect the efficiency of treatment; for example, a study conducted by Zhu *et al*. [48], injection of 1×10^6^ MSCs significantly reduced liver injury by concanavalin, whereas that of a low number MSCs (2×10^5^) had no effect. Repeated injection of MSCs for three times significantly improved survival and liver necrosis compared with single-dose infusion [50]. HLCs have multiple hepatic functions, such as promoting the renewal of multiple liver cells by lessening inflammation, necrosis, and apoptosis [42]. These inflammatory cells impair liver function and worsen inflammation-induced liver injury; therefore, MSC transplantation is directed to suppress these inflammatory cells and can help improve the sequelae of acute hepatitis [51]. In this study, the liver of the acute AFB1 control group showed swollen hepatomegaly with severe congestion and dark borders with a gross appearance with extensive necrosis of hepatocytes. The liver was restored to a nearly normal gross appearance and size after 4 weeks in the IV BM-MSCs and IH BM-MSCs groups, with few inflammatory cells infiltrating in IV BM-MSCs and apparently normal hepatocytes with few mononuclear cells infiltrating the portal area in IH BM-MSCs. In the IV HDCs group, the liver appeared swollen, congested, and enlarged and showed apparently normal hepatocytes. The IH HDCs group showed a nearly normal healthy gross appearance of the liver with the disappearance of congestion and Kupffer cell hyperplasia.

A general trend for the activities of the antioxidant enzymes (GSH-Px, CAT, and SOD) in hepatic tissue was observed. Acute AFB1 resulted in a decrease in the antioxidant enzyme activities compared with the healthy control group. IV therapy with BM-MSCs significantly increased the activities of CAT, SOD, and GSH-Px. IH therapy with BM-MSCs and IV therapy with HDCs did not ameliorate the toxic effect of AFB1. By contrast, IH therapy with HDCs significantly restored the activities of SOD and GSH-Px but did not lead to any change in CAT activity compared with the AFB1 control group. For the activities of the antioxidant enzymes in serum, GSH and TAC activities were significantly decreased in the acute AFB1 control group compared with those of the healthy control group. IV injection of BM-MSCs improved the activities of GSH and TCA. IH injection of HDCs was accompanied by a significant increase in serum GSH compared with the AFB1 control group.

In the acute AFB1 rat model, significant −ve correlations between GSH and TAC serum activities and TNF-α were observed. The same results were observed in rats treated with IV BM-MSCs, IH BM-MSCs, and IH HDCs. However, significant +ve correlations between GSH and TAC serum activity upregulation and TNF-α were found in IV HDCs rats. The antioxidant ability of BM-MSCs to ameliorate the bad effect of reactive oxygen species in tissues has been reported in humans [52] and in various animal models [53]. The possible mechanisms of protection were mainly through the secretion of vascular endothelial growth factor and NO [54], and elevation of SOD and MDA [55], and reduction of hepatocyte apoptosis *in vitro* and *in vivo* [56].

Regarding hepatic bio-indices, a significant decrease in ALB concentration and significant increase in ALT, AST, and NO were observed. IV injection of BM-MSCs significantly ameliorated ALT and AST activities. By contrast, IH injection of BM-MSCs significantly ameliorated ALT, AST, and NO compared with the acute AFB1 control group. IV therapy with HDCs did not affect the bio-index parameters and liver enzyme activities. Conversely, IH therapy with HDCs significantly increased ALB concentration and decreased ALT activity and NO concentration to approximately normal levels. These data are parallel with those of several previous works. BM-MSCs diminished liver transaminases (ALT and AST) and improved liver histopathology in mice with acute liver failure [57]. Transplantation of HLCs also ameliorated liver function by decreasing the expression of ALT, AST, and ammonia in mice with acute liver failure [58], CCl4-treated rats and mice [59,60], and rats with acetaminophen-induced acute liver failure [61]. In addition, MSCs significantly down-regulated the levels of liver transaminases and improved the histological images of mice with concanavalin A-induced hepatitis [62]. MSCs have also been found to reduce the serum concentrations of ammonia, ALT, AST, and total bilirubin in acute hepatitis animal models [37]. MSCs and HLCs upgraded liver function and saved CCl4-treated mice with liver damage by engraftment into the injured liver compared with the infusion of the liver with primary hepatocytes [63]. HLCs derived from MSCs significantly decreased liver dysfunction and enhanced the expression of ALB [64]. However, Brückner *et al*. [65] reported that HLCs decreased the portal venous pressure but had no effect on the activation of HSCs. In addition, Liu *et al*. [66] found that MSCs decreased the expression of transaminases, TNF-α and IL-6 and increased the expression of GSH and SOD. Transplantation through the splenic vein significantly decreased the levels of serum liver enzymes and increased the number of engrafted MSCs in the liver [43].

These data are in congruence with our finding that IV injection of HDCs and IH injection of MSCs yield similar results. We concluded that acute toxicity does not cause any prominent alteration due to its short duration. Therefore, the administration routes of MSCs, whether differentiated or not, do not significantly differ. In our opinion, acute toxicity can be treated through a non-invasive IV route without hepatogenic differentiation.

Regarding hepatic bio-indices and their correlations with TNF-α, in the acute AFB1 group, significant +ve correlations were observed between Chol, ALP, and TG upregulation and TNF-α. After treatment with IV BM-MSCs, IH BM-MSCs, and IH HDCs, significant −ve correlations were observed between Chol, TG, and NO serum levels and TNF-α. After treatment with IV and IH BM-MSCs, significant +ve correlations between ALT, AST, and ALP serum levels and TNF-α were recorded. After IV HDCs therapy, only a significant +ve correlation between ALT and AST serum level upregulation and TNF-α was observed, whereas a significant −ve correlation between ALP serum level and TNF-α was observed. After IH HDCs therapy, ALT and ALP serum upregulation showed significant +ve correlations with TNF-α but AST and TNF-α exhibited a significant negative correlation. Liver function is significantly improved after HLCs transplantation in mice with acute liver injury by stimulating anti-inflammatory cytokine secretion [49]. HLC transplantation in a partially hepatectomy-induced acute liver injury model significantly reduced lipid accumulation and restored liver capacity, thereby enhancing hepatocyte survival, preventing apoptosis, and eventually prolonging the survival of the animals [67]. HDCs transplantation also significantly reduced liver fibrosis by upregulating the expression of HGFs and lowering the serum levels of fibronectin and hepatic AFP [68]. Although HDCs have been proven to be effective in repairing liver injury in various diseases, the undifferentiated MSCs could be more effective because of the higher sensitivity of HDCs to *in vitro* and *in vivo* harsh environments [1].

The echogenicity of liver conditions refers to the transmission of USG waves in relation to surrounding tissues. Based on echogenicity, a structure can be characterized as hyperechoic; (white on the screen), hypoechoic; (gray), or anechoic (black). This study revealed that normal groups had homogeneous hepatic parenchyma with medium-level echogenicity and a straight hepatic surface, as previously described by Lessa *et al*. [69] and Mannheimer *et al*. [70]. The results of normal blood flow parameters using USG are also parallel with those of a previous study of Sonhaye *et al*. [71], and the finding on abnormal blood flow due to acute hepatitis is comparable with that of previous study of Lessa *et al*. [69]. Some images of acute hepatitis appeared to be normal in USG examination, which is also comparable with data reported in the study of Palmentieri *et al*. [72], indicating that USG has a high sensitivity for the detection of marked hepatitis or the presence of fibrosis but that its sensitivity decreases when hepatitis is mild or acute.

## Conclusion

BM-MSCs and their derivatives, including HDCs can be used for acute AF liver injury repair in individuals or animal models by grafting into liver tissue, differentiating and secreting anti-inflammatory and antioxidant factors, and enhancing *in vivo* liver regeneration and immunomodulation. Furthermore, the administration routes of BM-MSCs did not demonstrate any significant difference; however, IH route of HDCs showed significant amelioration than IV route. Due to the sole effect of TNF-α as a prominent inflammatory mediator: correlated with most measured data, thereby providing a general picture of amelioration in the form of +ve or −ve correlation with up or down-regulation. Therefore, it can be concluded that MSCs and HDCs are useful tools in regenerative medicine that target acute liver injury and toxicity. However, acute hepatitis can be treated by a non-invasive IV route without the expense of hepatogenic differentiation. Further research using clinical trials that address several problems regarding engraftment and potentiation is needed to determine the optimal manipulation strategy in animal models and achieve better long-term effects with low doses of drugs.

## Authors’ Contributions

FAMA and AAZ: Designed the study. FAMA: Cultured rat BM-MSCs and examined their viability and phenotypic analysis and proliferation. FAMA: Differentiated and characterized hepatogenic cells. AAZ, RMA, and AF: ConductedRT-PCR. FAMA, AAZ, and AF: Determined metabolic activity of HDCs. FAMA: Managed USG examination. RMA: Determined cytokine profile. FAMA, RMA, and AF: Measured hepatic bio-indices and serum antioxidant activities. AF: Histological assay. AAZ: Interpretation of data and statistical analysis. FAMA and AAZ: Drafted the manuscript. All authors read and approved the final manuscript.
